# Are admissions for gout preventable? An audit

**DOI:** 10.1186/1471-2474-14-S1-A12

**Published:** 2013-02-14

**Authors:** Anetha Sabanathan, John Dillon, Lesley Hordon

**Affiliations:** 1 Dewsbury District Hospital, Dewsbury, WF13 4HS, UK

## Introduction

The number of ward referrals due to gout has increased. Gout can result in a prolonged admission. Guidelines already exist for gout treatment [1].

The aim of the audit is:-

1. To identify preventable factors in admissions/hospital flares of gout.

2. To estimate length of stay due to gout.

## Methods

The notes of 50 consecutive inpatients referred to Rheumatology with gout over a 28 month period were reviewed.

## Results

32 (64%) primarily admitted for gout with a median stay of 8 days. 6 of these admissions were due to polyarticular gout. 18 patients had gout developing during an admission for another cause. Length of stay was increased in 4 of these patients by a median of 4.5 days. Of those primarily admitted with gout, 17 had a past history of gout, of which 13 had either been on or were still taking allopurinol prior to admission. Of those that developed gout during admission, 11 had a past history of gout with 4 having been on or still on allopurinol. All gout flares were treated with either NSAID (15) or colchicine (35). Of those that had steroid treatment (13), NSAIDs or colchicine were used appropriately. 14 had antibiotic treatment for joint swelling, 3 prior to joint aspiration [2]. Of those with a past history of gout 29, 17 had a history of past allopurinol use prior to admission, at least 7 had more than one episode per year, 5 of these 7 had a prior history of allopurinol.

Of 17 patients previously treated with allopurinol, 6 had discontinued the drug prior to admission. Of those that had stopped allopurinol, at least 3 had a flare of their gout within 3 months of stopping. Of those who were on allopurinol prior to admission, the dose was only 100 mg.

## Discussion

Total estimated number of bed days due to gout over study period was 349 days. A potential of 28 patient admissions were preventable. It was estimated that 2 patients could have had admissions/flares prevented if they had received allopurinol or other uric acid lowering drug prior to gout admission/flare according to BSR guidelines. It is estimated 6 patients inappropriately stopped their allopurinol. A further 11 were on inappropriately low doses of allopurinol.

The majority had the acute episode treated appropriately.

## Conclusion

At least 19 (38%) gout flares out of the total 50 patients reviewed were preventable, with a maximum potential of 28 (56%) flares being preventable.
